# Biologic and targeted synthetic DMARD safety in inflammatory arthritis: British Society for Rheumatology guideline scope

**DOI:** 10.1093/rap/rkaf104

**Published:** 2025-10-14

**Authors:** Stephanie F Ling, Joshua L Bennett, Vanessa J Reid, Saad Ahmed, Mostafa Meshaal Ahmad, Sarah Bennett, Marwan Bukhari, Tom Carver, Mary Chatten, Samantha Cooray, Lucy Craig, Cathy Donaghy, James Galloway, Mark Gibson, Rebecca Heaton, Holly Henderson, Nicola Holroyd, Akhila Kavirayani, Alison Kent, Mariam Malik, Andrew Melville, Tanjina Nasrin, Adlan Wafi Ramli, Alan Rawlings, Emily Rose-Parfitt, Luke Sammut, Ethan S Sen, Demisha Vaghela, Angela Vint, Rosemary Waller, Emma L Williams, Taryn Youngstein, Jean Zhang, Sizheng Steven Zhao, Christopher Holroyd, British Society for Rheumatology Guideline Steering Group, Stephanie F Ling, Joshua L Bennett, Vanessa J Reid, Saad Ahmed, Mostafa Meshaal Ahmad, Sarah Bennett, Marwan Bukhari, Tom Carver, Mary Chatten, Samantha Cooray, Lucy Craig, Cathy Donaghy, James Galloway, Mark Gibson, Rebecca Heaton, Holly Henderson, Nicola Holroyd, Akhila Kavirayani, Alison Kent, Mariam Malik, Andrew Melville, Tanjina Nasrin, Adlan Wafi Ramli, Alan Rawlings, Emily Rose-Parfitt, Luke Sammut, Ethan S Sen, Demisha Vaghela, Angela Vint, Rosemary Waller, Emma L Williams, Taryn Youngstein, Jean Zhang, Sizheng Steven Zhao, Christopher Holroyd

**Affiliations:** Centre for Musculoskeletal Research, University of Manchester, Manchester, UK; Paediatric Rheumatology Department, Great North Children’s Hospital, Newcastle-upon-Tyne, UK; Kellgren Centre for Rheumatology, Manchester University NHS Foundation Trust, Manchester, UK; Department of Rheumatology, Cambridge University Hospital, Cambridge, UK; Department of Rheumatology, NHS Fife, Kirkcaldy, UK; Rheumatology Department, University Hospital Southampton NHS Foundation Trust, Southampton, UK; Department of Rheumatology, University Hospitals of Morecambe Bay NHS Foundation Trust, Lancaster, UK; Expert by Experience, Wirral, UK; Department of Paediatric Ophthalmology, Queens Medical Centre, Nottingham, UK; Department of Rheumatology, Queens Medical Centre, Nottingham, UK; Department of Paediatric Rheumatology, Great Ormond Street Hospital, London, UK; Paediatric Rheumatology Department, Great North Children’s Hospital, Newcastle-upon-Tyne, UK; Department of Rheumatology, Belfast Health and Social Care Trust, Belfast, UK; Centre for Rheumatic Diseases, King’s College London, London, UK; Centre for Rheumatic Diseases, King’s College London, London, UK; Department of Rheumatology, Stepping Hill Hospital, Stockport NHS Foundation Trust, Stockport, UK; Expert by Experience, Bristol, UK; North Baddesley Surgery, Southampton, UK; Department of Paediatric Rheumatology, Oxford University Hospitals NHS Trust, Oxford, UK; Rheumatology Department, Salisbury NHS Foundation Trust, Salisbury, UK; Rheumatology Department, Hampshire Hospitals NHS Foundation Trust, Basingstoke, UK; Department of Rheumatology, York and Scarborough Teaching Hospitals NHS Foundation Trust, York, UK; School of Infection and Immunity, University of Glasgow, Glasgow, UK; Rheumatology Department, Salisbury NHS Foundation Trust, Salisbury, UK; Department of Acute Medicine, Royal Liverpool University Hospital, Liverpool, UK; Expert by Experience, Stone, UK; Department of Rheumatology, North Bristol NHS Trust, Bristol, UK; Department of Rheumatology, Portsmouth Hospitals University NHS Trust, Portsmouth, UK; Paediatric Rheumatology Department, Great North Children’s Hospital, Newcastle-upon-Tyne, UK; Faculty of Medical Sciences, Newcastle University, Newcastle-upon-Tyne, UK; Department of Children’s Rheumatology, University Hospitals of Leicester NHS Trust, Leicester, UK; Expert by Experience, Ludlow, UK; Rheumatology Department, Great Western NHS Foundation Trust, Swindon, UK; Rheumatology Department, Hampshire Hospitals NHS Foundation Trust, Winchester, UK; Department of Rheumatology, Imperial College Healthcare NHS Trust, London, UK; Rheumatology Department, University Hospital Southampton NHS Foundation Trust, Southampton, UK; Centre for Musculoskeletal Research, University of Manchester, Manchester, UK; Rheumatology Department, University Hospital Southampton NHS Foundation Trust, Southampton, UK

**Keywords:** Rheumatoid arthritis, psoriatic arthritis, axial spondyloarthritis, juvenile arthritis, biological therapy, DMARD, JAK inhibitor, safety, guideline

## Abstract

This guideline will provide up-to-date and evidence-based recommendations for the safe use of biologic and targeted synthetic DMARDs in individuals with inflammatory arthritis (IA) across the life course. Important updates from the 2019 iteration of this guideline will include the incorporation of newer pharmacotherapies (such as Janus kinase inhibitors) and an extension of the target clinical population to cover children and young people with IA. The guideline will be updated and produced in accordance with the British Society for Rheumatology protocol for developing clinical guidelines, updated 2023.

## Introduction

The armamentarium available to treat inflammatory arthritis (IA) continues to expand. In addition, there is increasing recognition that people with IA are often affected by multiple long-term conditions [1], adding complexity to the safe prescription of targeted therapies. In 2019, the safety guideline for the use of biologic DMARDs (bDMARDs) in adults with IA was produced by the British Society for Rheumatology (BSR) [2]. The guideline covered the use of the TNF inhibitors (TNFis) abatacept, rituximab, tocilizumab and ustekinumab for the treatment of RA, PsA and axial SpA (axSpA, including AS). Therapies approved for these indications by National Institute for Health and Care Excellence after June 2016 were not included.

## Why the updated guideline is needed

Since the publication of the existing guideline, several new classes of biologic therapies have become available, including IL-17 inhibitors (bimekizumab, ixekizumab and secukinumab) and IL-23 inhibitors (guselkumab and risankizumab). Furthermore, a new class of targeted therapy, Janus kinase inhibitors (JAKis), has now been approved for the treatment of IA. These small molecules (baricitinib, filgotinib, tofacitinib and upadacitinib) are classed as targeted synthetic DMARDs (tsDMARDs). Concerns regarding the safety of JAKis have resulted in the Medicines and Healthcare products Regulatory Agency (MHRA) and the European Medicines Agency (EMA) issuing guidance recommending caution with their use in certain populations, such that their use should be limited to individuals without risk factors for cardiovascular disease (CVD), venous thromboembolism (VTE) and malignancy [3].

The existing 2019 guideline provided safety recommendations for the use of bDMARDs in adults with IA, but no equivalent guidance exists for children and young people (CYP) with JIA; many of these individuals continue to require specialist care and b/tsDMARDs into adulthood [4, 5]. Although not all guidance for adults with IA will be applicable to CYP with JIA and vice versa, we anticipate that many of the principles concerning the safe prescribing of b/tsDMARDs for IA will be relevant throughout the life course, particularly given that many medications included in the updated guideline are used by both CYP and adults with IA. The drugs anakinra (anti-IL-1) and canakinumab (anti-IL-1β), which are not usually used to treat adult IA (excepting adult-onset Still’s disease), but can be used in CYP with JIA, will therefore also be included in this new guideline.

Henceforth ‘IA’ will be used to refer to JIA, Still’s disease across the life course [incorporating systemic JIA), RA, PsA and seronegative SpA (including axSpA, AS and non-radiographic axSpA (nr-axSpA)].

## Objectives

Our objectives are to inform the rheumatology community that the 2019 BSR bDMARD safety guideline for IA is in the process of being updated, to clarify the intended remit of the updated guideline and to share the methodology for updating the existing guideline and facilitate transparency for when the final updated guideline is published.

## Who the guideline is for

The updated guideline is intended for use by healthcare professionals who are involved in the management of people with IA receiving b/tsDMARDs. This may include doctors, nurses, pharmacists or allied healthcare professionals working with adults or CYP with IA. In addition to healthcare professionals working in rheumatology departments, the updated guideline may be informative for general practitioners (GPs), physicians in other specialties and surgeons managing people with IA treated with b/tsDMARDs. Finally, we intend that the updated guideline will be a useful resource for people and families affected by IA as well as other interested parties such as organisations for people with IA.

## What the guideline will cover

### Target clinical population

The target clinical population is people of all ages (adults, CYP) with IA, as defined above.

### Settings

Settings include primary, secondary and tertiary care, especially adult and paediatric rheumatology departments.

### Key areas that will be covered

Key areas that will be covered include:

- Updated recommendations on the use of licensed and available b/tsDMARDs, including JAKis
- New recommendations on b/tsDMARD prescribing choices following screening for the risk of drug-induced lupus, infections, malignancy, HIV, VTE and CVD
- Updated recommendations on b/tsDMARD prescribing choices for people with IA with concurrent CTD–interstitial lung disease (ILD)
- Updated recommendations on b/tsDMARD prescribing choices for people with IA who develop uveitis, psoriasis and IBD while on drug
- Updated guidance on management of people with IA receiving b/tsDMARDs during the perioperative period
- Updated guidance on the timing of vaccination intervals for people with IA receiving b/tsDMARDs, including those for CYP with IA.

### Areas that will not be covered

Areas that will not be covered include:

- The use of conventional synthetic (cs)DMARDs or apremilast, which are included in the upcoming BSR csDMARD in IA safety guideline [6]
- Indications for b/tsDMARD therapy and assessment of b/tsDMARD efficacy
- Prescribing in relation to pregnancy and breastfeeding, as there is a separate BSR guideline for this [7]
- The use of b/tsDMARDs in conditions other than IA, as defined in the Target Clinical Population section
- Specific guidance for biosimilar preparations; recommendations from this guideline apply to both originator and biosimilar preparations.

## Methodology

The existing guideline will be updated in accordance with BSR’s protocol for creating clinical guidelines [8].

In 2024, the existing bDMARD safety guideline from 2019 [2] was reviewed by the current BSR Guideline Working Group in the format of an online survey, with the aim of identifying recommendations that require updating or for which new evidence has emerged. Survey responses aided drafting of the key issues and questions below and will also inform updated recommendations in the upcoming guideline document.

A systematic literature review will be carried out to identify new studies not included in the previous guideline that offer insights into b/tsDMARD treatment, monitoring and safety. A panel of clinical, academic and experiential experts will then review the gathered evidence and produce a draft guideline. A public and professional consultation will then commence, where the draft guideline will be available for consultation among healthcare professionals and organisations for people with IA to ensure it meets clinical needs. Feedback will be incorporated into this guideline and it will be published once finalised.

## Time frame

The guideline is expected to be published in 2026/2027.

## Related guidance

Where related guidance from other expert clinical organisations exists, the updated b/tsDMARD guideline will incorporate recommendations to prevent duplication of literature synthesis. Related guidance includes:

- 2014 Childhood Arthritis and Rheumatology Research Alliance (CARRA) consensus treatment plans for new-onset polyarticular JIA [9]
- 2019 BSR bDMARD safety guideline in IA [2]
- 2021 ACR guideline for the treatment of JIA: recommendations for non-pharmacological therapies, medication monitoring, immunisations and imaging [10]
- 2021 ACR guideline for the therapeutic treatment of JIA: oligoarticular, TM joint and systemic subtypes [11]
- 2021 EULAR recommendations for patient self-management in IA [12]
- 2021 EULAR/Paediatric Rheumatology European Society (PReS) recommendations for vaccination of paediatric patients with autoimmune inflammatory rheumatic diseases [13]
- 2022 ACR guideline for vaccinations in patients with rheumatic and musculoskeletal diseases [14].
- 2022 ACR/American Association of Hip and Knee Surgeons guideline for the perioperative management of anti-rheumatic medication in patients with rheumatic diseases undergoing total hip or knee arthroplasty [15].
- 2022 Assessment of SpondyloArthritis international Society (ASAS)-EULAR recommendations for management of axSpA [16]
- 2022 EULAR recommendations for b/cs/tsDMARD use in RA [17]
- 2022 EULAR recommendations for screening and prophylaxis of chronic and opportunistic infections in adults with autoimmune inflammatory rheumatic diseases [18]
- 2023 BSR guideline on prescribing immunomodulator drugs and corticosteroids in pregnancy and breastfeeding [7]
- 2023 EULAR consensus statement on IL-6 inhibition in inflammatory conditions [19]
- 2023 EULAR recommendations for pharmacological management of PsA [20]
- 2024 EULAR/PReS recommendations for diagnosis and management of Still’s disease throughout the life course [21]
- 2024 EULAR points to consider on the initiation of targeted therapies in patients with inflammatory arthritis and a history of cancer [22]
- 2025 BSR guideline for the treatment of axSpA with b/tsDMARDs [23]
- Immunisation against infectious disease: the Green Book [24]
- UK Clinical Pharmacy Association Handbook of Perioperative Medicines [25].

## Key issues and questions

We identified the following draft PICO (patients/population/problem, intervention, comparison and outcome) statements to direct the systemic literature review. Evidence from clinical trials and real-world observational studies will be included, where available. Where relevant, existing guidance from organisations such as EULAR will be acknowledged and updated. We acknowledge that there is likely to be a paucity of evidence in some areas, particularly for CYP populations, and that it might not be possible to make recommendations in all areas.

Some recommendations will be made in the absence of a dedicated PICO in the following situations: expert opinion from the Guideline Working Group felt that there is a high likelihood of a very weak evidence base, recent other guidelines have covered the topic or there is a high likelihood that the evidence base has not changed from the previous guideline. A smaller updated literature review may be undertaken for any recommendations that were not covered by a specific PICO to ensure this guideline is up to date.

### PICO statement 1 proposal

Population (P): People with IA due to commence a first TNFi.

Intervention (I): Check ANA during pre-drug screening.

Comparison (C): Not checking ANA.

Outcome (O): Rate of drug-induced lupus-like syndrome with TNFi bDMARDs.

### PICO statement 2 proposal

Population (P): People with IA due to commence a b/tsDMARD at high risk for infection (e.g. poorly controlled diabetes, pre-existing heart or lung disease, multiple recurrent infections, recent chemotherapy/radiotherapy, primary immunodeficiency, long-term glucocorticoid use).

Intervention (I): Choose agent with superior infection profile preferentially.

Comparison (C): Follow usual local prescribing guideline, regardless of infection risk status.

Outcome (O): Rate of infections.

### PICO statement 3 proposal

Population (P): People with IA due to commence a b/tsDMARD with a known premalignant condition.

Intervention (I): Prescribe personalised choice of b/tsDMARD, due to increased risk of malignancy.

Comparison (C): Follow usual local prescribing guideline, regardless of malignancy risk status.

Outcome (O): Rate of malignancy.

### PICO statement 4 proposal

Population (P): People with IA with associated ILD (CTD-ILD) due to commence a b/tsDMARD.

Intervention (I): Prescribe personalised choice of b/tsDMARD to more effectively treat IA and ILD.

Comparison (C): Follow usual local prescribing guideline, regardless of ILD status.

Outcome (O): Pulmonary outcomes.

### PICO statement 5 proposal

Population (P): People with IA on etanercept who go on to develop uveitis while on-drug.

Intervention (I): Switch to an alternate b/tsDMARD.

Comparison (C): Continue on etanercept.

Outcome (O): Incidence and rate of recurrence of uveitis.

### PICO statement 6 proposal

Population (P): People with IA due to commence a b/tsDMARD.

Intervention (I): Screen for VTE risk factors during pre-drug screening and prescribe personalised choice of b/tsDMARD, based on risk.

Comparison (C): Follow usual local prescribing guideline, regardless of VTE risk status.

Outcome (O): Rate of VTE.

### PICO statement 7 proposal

Population (P): People with IA due to commence a b/tsDMARD.

Intervention (I): Screen for CVD risk factors during pre-drug screening and prescribe personalised choice of b/tsDMARD, based on risk.

Comparison (C): Follow usual local prescribing guideline, regardless of CVD risk status.

Outcome (O): Rate of CVD.

### PICO statement 8 proposal

Population (P): People with IA on a b/tsDMARD undergoing surgery.

Intervention (I): Stopping b/tsDMARD for one dosing interval preoperatively (in elective procedures).

Comparison (C): Continue b/tsDMARD as usual.

Outcome (O): Rate of postoperative complications.

### PICO statement 9 proposal

Population (P): People with IA on a b/tsDMARD undergoing surgery (includes both elective and emergency).

Intervention (I): Delaying restart of b/tsDMARD until risk of postoperative infection is reduced and satisfactory wound healing has been achieved (typically ≈14 days after surgery).

Comparison (C): Continue b/tsDMARD as usual.

Outcome (O): Rate of postoperative complications.

### PICO statement 10 proposal

Population (P): People with IA receiving an IL-17 inhibitor who go on to develop IBD on-drug.

Intervention (I): Switch to an alternate b/tsDMARD.

Comparison (C): Continue on IL-17 inhibitor.

Outcome (O): Incidence and rate of recurrence of IBD.

### PICO statement 11 proposal

Population (P): People with IA due to commence a b/tsDMARD or already receiving a b/tsDMARD.

Intervention (I): Interrupt usual vaccination schedule (as per Green Book).

Comparison (C): Continue usual vaccination schedule.

Outcome (O): Rates of vaccine immunogenicity (cellular and serological response) and of infections covered by vaccination schedule.

## Guideline working group

Christopher Holroyd (chair, rheumatologist), Mostafa Meshaal Ahmad (rheumatologist), Saad Ahmed (rheumatology trainee), Joshua L Bennett (paediatric rheumatology trainee), Sarah Bennett (rheumatology nurse), Marwan Bukhari (rheumatologist), Tom Carver (expert by experience), Mary Chatten (rheumatology nurse), Samantha Cooray (paediatric rheumatology trainee), Lucy Craig (paediatric rheumatology nurse), Cathy Donaghy (rheumatologist), James Galloway (rheumatologist), Mark Gibson (rheumatology trainee), Rebecca Heaton (rheumatology pharmacist), Holly Henderson (expert by experience), Nicola Holroyd (GP), Akhila Kavirayani (paediatric rheumatologist), Alison Kent (rheumatology nurse), Stephanie F. Ling (rheumatologist), Mariam Malik (rheumatologist), Andrew Melville (rheumatology trainee), Tanjina Nasrin (rheumatology trainee), Adlan Wafi Ramli (rheumatologist), Alan Rawlings (expert by experience), Vanessa J. Reid (rheumatology pharmacist), Emily Rose-Parfitt (life course rheumatology pharmacist), Luke Sammut (rheumatologist), Ethan S. Sen (paediatric rheumatologist), Demisha Vaghela (paediatric rheumatology pharmacist), Angela Vint (expert by experience), Rosemary Waller (life course rheumatologist), Emma L. Williams (rheumatologist), Taryn Youngstein (rheumatologist), Jean Zhang (rheumatology trainee), Sizheng Steven Zhao (rheumatologist).

## Supplementary Material

rkaf104_Supplementary_Data

## Data Availability

No new data were generated or analysed in support of this document.
