# Supplementary material for: Biologic and targeted synthetic DMARD safety in inflammatory arthritis: British Society for Rheumatology guideline scope
Source: Rheumatol Adv Pract. 2025 Oct 14;9(4):rkaf104. doi: 10.1093/rap/rkaf104 (PMC12517738; doi:10.1093/rap/rkaf104)
Supplement: rkaf104_Supplementary_Data [file rkaf104_supplementary_data.zip › Supplementary Data S1.docx]

**Supplementary Data S1. British Society for Rheumatology Guideline Steering Group members**

Abhishek Abhishek, Anoop Kuttikat, Arvind Kaur, Christopher Joyce, Claire Jones, Coziana Ciurtin, Devesh Mewar, Edward Roddy, Emily Rose-Parfitt, Emma Williams, Emmandeep Dhillon, Hirushi S. Jayasekera, Karen Merrison, Pratyasha Saha, Sandrine Compeyrot-Lacassagne.
